# Serum Fatty Acid-Binding Protein 4 is Increased in Patients with Psoriasis

**DOI:** 10.1007/s11745-016-4211-4

**Published:** 2016-11-18

**Authors:** A. Baran, M. Świderska, Joanna Bacharewicz-Szczerbicka, H. Myśliwiec, I. Flisiak

**Affiliations:** 1 Department of Dermatology and Venereology, Medical University of Bialystok, Zurawia 14 St, 15-540 Bialystok, Poland; 2 Department of Infectious Diseases and Hepatology, Medical University of Bialystok, Zurawia 14 St, 15-540 Bialystok, Poland

**Keywords:** Psoriasis, Fatty acid-binding protein 3, Fatty acid-binding protein 4, Anthralin, Lipids, Metabolic syndrome

## Abstract

Psoriasis is associated with metabolic syndrome and cardiovascular disease. Fatty acid-binding proteins (FABP) have been recognized as predictors of these systemic disorders. The aim of this study was to assess correlations between levels of serum heart and adipocyte fatty acid-binding proteins (FABP3, FABP4) and disease severity, indicators of inflammation or metabolic disturbances, and topical treatment in psoriatic patients. Thirty-seven patients with relapse of plaque-type psoriasis and 16 healthy volunteers were recruited. Blood samples were collected before and after 14 days of therapy. Serum FABP concentrations were examined by enzyme-linked immunosorbent assay for correlation with Psoriasis Area and Severity Index (PASI), body mass index (BMI), inflammatory or metabolic parameters, and treatment used. The median FABP4 serum levels were significantly increased (*p* = 0.038) in psoriatic patients, while FABP3 levels did not differ (*p* = 0.47) compared to the controls. No significant correlations were noted between the proteins and PASI, C-reactive protein (CRP), BMI, or levels of glucose or lipids. FABP3 significantly correlated with white blood count (*p* = 0.03) and aspartate aminotransferase (*p* = 0.04). After topical treatment, there was no significant change in serum FABP3 [11.5 (4.9–30.3) vs. 12.9 (3.5–30.3) ng/ml] (*p* = 0.96), whereas FABP4 was decreased [27,286 (20,344–32,257) vs. 23,034 (18,320–29,874) pg/ml] (*p* = 0.12), losing its basal significance. FABP4 may be a marker of psoriasis, and FABP3 may be associated with inflammation or liver disorders in psoriatic patients. FABP do not appear to be useful for determining disease severity or the effectiveness of antipsoriatic treatment.

## Introduction

Psoriasis is a chronic inflammatory dermatosis with a multifactorial pathogenesis, affecting over 2% of the world’s population. Recently it has been considered as a systemic disorder closely associated with coronary artery disease (CAD), hypertension, diabetes mellitus (DM), obesity, metabolic syndrome (MS) and atherosclerosis [1, 2]. Psoriatic patients live on average 5 years less than the general population, which is due to increased risk of myocardial infarction (MI) and thromboembolic events [3]. The risk of developing DM, obesity and MS is more than double, occurring in over 40% of psoriatic patients [4]. Common links between psoriasis and its comorbidities mainly involve the chronic inflammation that emerges from metabolic tissues (metaflammation), genetic basis, similar pathways of immune disorders, and bioactive substances synthesized and secreted by adipose tissue [4–6]. Numerous published studies, as well as ours, have demonstrated the potential role of various adipokines in the development of psoriasis and its comorbidities [5, 7].

Lipids, such as fatty acids (FA) and their derivatives, perform various biological functions, including their role in energy homeostasis, the formation of substrates for cell membranes, and signaling molecules in inflammatory and metabolic pathways [8]. Disturbances in lipid homeostasis can lead to numerous common disorders including obesity, insulin resistance (IR), DM, cardiovascular disease, and even skin disorders [9, 10]. Fatty acids, which constitute about 15% of lipids in the stratum corneum, participate in maintaining the permeability of the epidermis, for example, by stimulating acidification of the horny layer. Research has shown that deviations in FA composition in keratinocytes may be involved in the pathogenesis of inflammatory dermatosis such as atopic dermatitis or psoriasis [11]. FA shifted to long-chain fatty acids (LCFA) are transported by certain proteins to various tissues, where they are metabolized, stored or utilized [9]. Fatty acid-binding proteins (FABP), first identified in 1972, are a 14–15 kDa family of cytosol proteins involved in active regulation of lipid trafficking and the protection of organisms against harmful accumulation of LCFA [8, 10, 11]. To date, at least nine isoforms have been identified, and they have been named based on the tissues in which they are prominently expressed or those that actively participate in lipid metabolism: liver, intestines, heart, adipose tissue, epidermis, ileus, brain, myelin and testis. FABP isoforms are thought to be useful indicators of organ damage and to play an important role in the development of a number of systemic diseases. FABP1 (liver FABP), present mainly in hepatocytes and excreted by the kidneys, indicates acute kidney damage and liver disorders, while the ileal isoform may be associated with ileitis or IR [10].

Epidermal fatty acid-binding protein 5 (FABP5), with its gene located at chromosome 8q21.13, is mostly expressed in epidermal cells, but also in other tissues including the brain, kidney, liver, lungs, and testis and in adipose tissue. Various studies have stressed its role in regulating insulin sensitivity, lipid homeostasis, MS, skin barrier conditions, and even nerve regeneration [8, 10, 12, 13]. FABP5 is also called psoriasis-associated FABP (PA-FABP), which reflects its already proven links with this dermatosis [12, 13]. Some studies have indicated that FABP5 modulates the differentiation of keratinocytes and is highly over-expressed in psoriatic plaques or atopic dermatitis [12, 13].

Heart-type FABP (FABP3), with the encoding gene located on chromosome 1p33–p31, is expressed mainly in the heart, skeletal muscle, brain, mammary glands and brown adipose tissue. FABP3 is superior to troponin as a highly sensitive marker of acute myocardial infarction and predictor of heart failure in patients with MS, which is strongly associated with psoriasis [8, 10]. FABP3 also participates in thermoregulation and affects glucose homeostasis and cardiac sufficiency [9, 10]. A precise role of FABP3 in other tissues and the development of certain diseases is still largely unknown.

Adipocyte fatty acid-binding protein (FABP4), also termed adipocyte protein 2 (aP2), is an adipokine synthesized and released predominantly from adipocytes and, to a lesser extent, produced in macrophages and endothelial cells [6, 14]. FABP4, with a molecular mass of 14.6 kDa and 132 amino acids, accounts for up to 6% of total cellular proteins. The gene encoding this adipokine is located on chromosome 8q21. Elevated levels of FABP4 are closely linked with the development of obesity, IR, DM, hypertension, CAD and atherosclerosis [10, 14, 15]. Studies in humans have shown that a genetic variant at the FABP4 locus reduced the risk for atherosclerosis, diabetes and coronary heart disease [16, 17], and reduced FABP4 expression in adipose tissue was related to lower risk for CAD and DM [16]. Several studies have proved that increased circulating FABP4 levels are a promising biomarker predicting heart failure, stroke, and prognosis and mortality in patients with end-stage renal disease, CAD or other critical illnesses [6, 14, 15, 20, 21].

Another possible role of FABP4 in psoriasis is evident in the influence on MS, which is strongly associated with psoriasis. FABP4 is an independent predictive marker for the development of MS, as was shown in a 5-year prospective study and in a study of Korean boys [18, 19].

A further link between FABP4 and psoriasis may be a correlation with tumor necrosis factor-α (TNF-α), which is one of the major cytokines involved in the pathogenesis of this dermatosis. FABP4-deficient macrophages and treatment with TNF-α inhibitors was found to lead to a significant reduction in cytokine levels [14]. FABP4 has been linked to angiogenesis and vascular endothelial growth factor (VEGF), which are also highly disturbed in psoriasis [6].

In summary, FABP4 and FABP3 may play a pivotal role in numerous disorders, including obesity, atherosclerosis, MS, IR and CAD, which are closely associated with psoriasis. The actual role of the FABP in the pathogenesis of the dermatosis and its metabolic comorbidities has not yet been investigated. The aim of this study, therefore, was to evaluate serum FABP4 and FABP3 levels in patients with plaque-type psoriasis, and the relationship between these FABP and the duration and activity of the disease as well as the inflammatory or metabolic markers, selected adipokines and changes after standard topical therapy. A second objective of the study was to assess whether FABP3 and FABP4 could be useful for predicting the risk of cardiovascular disorders or metabolic diseases in patients with psoriasis.

## Materials and Methods

In this prospective study, 37 patients (15 women and 22 men) aged 19–84 years (mean 48.6 ± 2.4) with flare of plaque-type psoriasis were recruited in the Department of Dermatology and Venereology at the Medical University of Bialystok. The exclusion criteria were other forms of psoriasis or any chronic inflammatory, autoimmune or metabolic diseases. None of the patients was under dietary restrictions, and no patients received any chronic systemic or topical treatment for 1 month prior to enrollment. The Psoriasis Area and Severity Index (PASI) score was determined by the same investigator in all patients, with PASI scoring as follows: <10 points indicated mild psoriasis; 10—20 indicated moderate psoriasis; >20 indicated severe disease. Body mass index (BMI) was calculated as weight/height^2^ (kg/m^2^). Blood samples were collected before initiation and after 2 weeks of topical treatment with 5% salicylic acid ointment and 0.3% anthralin. FABP4 and FABP3 serum concentrations were evaluated in relation to normal values collected from 16 healthy age-, sex- and BMI-matched volunteers. The study was approved by the bioethics committee of the Medical University of Bialystok, and all participants provided written informed consent before enrollment.

## Serum Collection

Blood samples were collected from the study and control groups using Vacutainer tubes, and were left for 30 min to allow clotting before centrifugation for 15 min at 1000*g*, after which the serum was separated and stored at −80 °C until use. FABP3 and FABP4 levels were measured using the Quantikine^®^ enzyme immunoassay kit (R&D Systems, Minneapolis, MN, USA). The limit of detection for FABP3 was 0.273 ng/ml and for FABP4 was 13.3 pg/ml, and the standard curve ranges were 0.625–40 ng/ml and 31.2–2000 pg/ml, respectively. Optical density was read at a wavelength of 450 nm. The concentrations were assessed by interpolation from calibration curves prepared with standard samples provided by the manufacturer.

## Statistical Analysis

Statistical analysis was performed using GraphPad Prism 5 (GraphPad Software, La Jolla, CA, USA) and Statistica 10.0 (StatSoft, Inc., Tulsa, OK, USA). A value of *p* < 0.05 was considered statistically significant. Data are presented as median (IQR, interquartile range), percentage and mean ± standard deviation (±SD) when appropriate. The following non-parametric (distribution-free) tests were applied: Mann–Whitney *U* test for comparisons between psoriasis and control groups, Kruskal–Wallis analysis of variance (ANOVA) for comparisons of multiple groups (PASI groups), and Spearman’s rank test for correlations analyses.

## Results

**Fig. 1:**
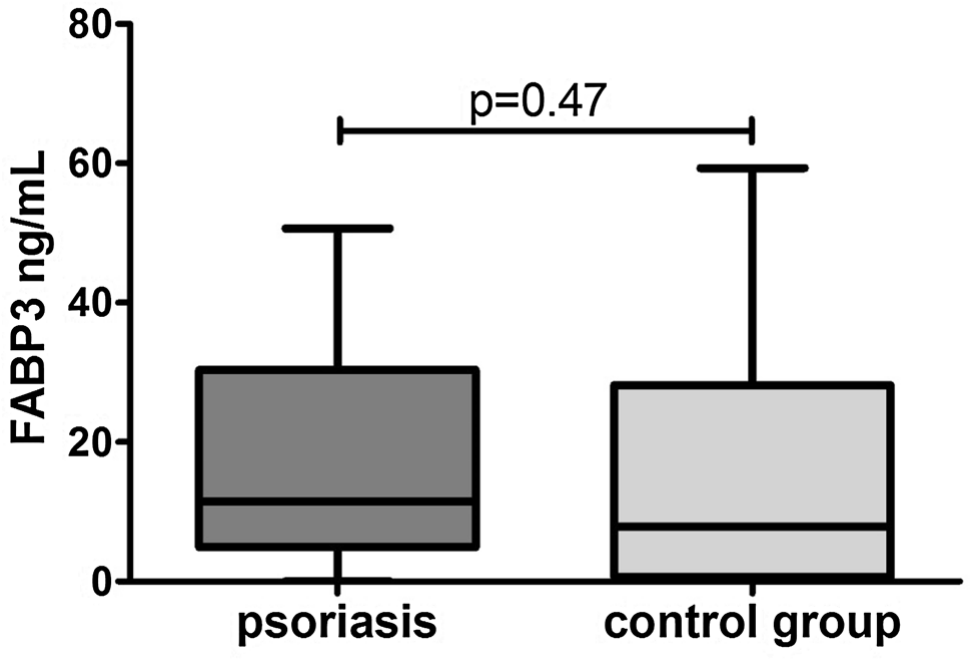
Comparison of serum FABP3 concentrations between patients and controls

**Fig. 2:**
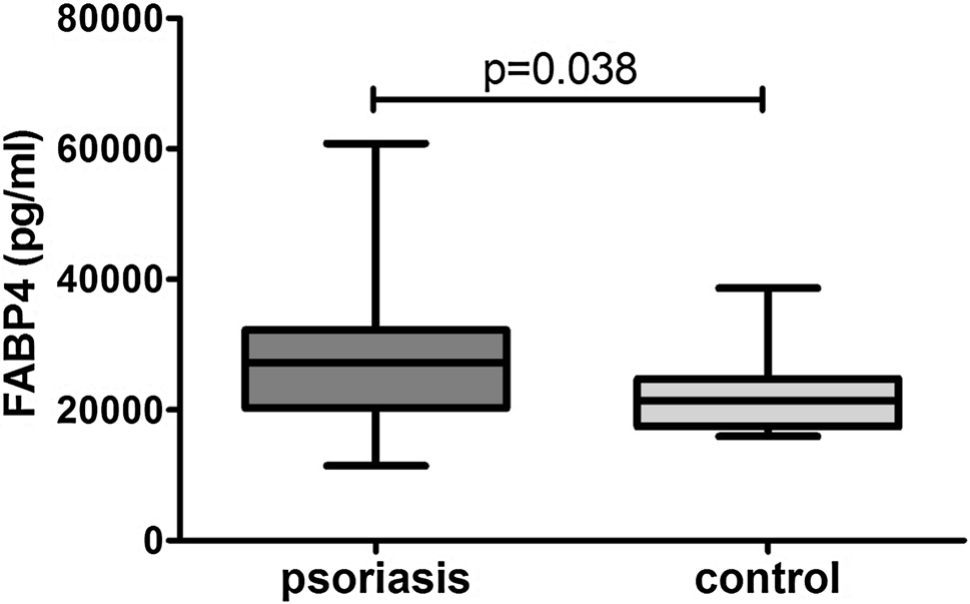
Comparison of serum FABP4 concentrations between patients and controls

**Fig. 3:**
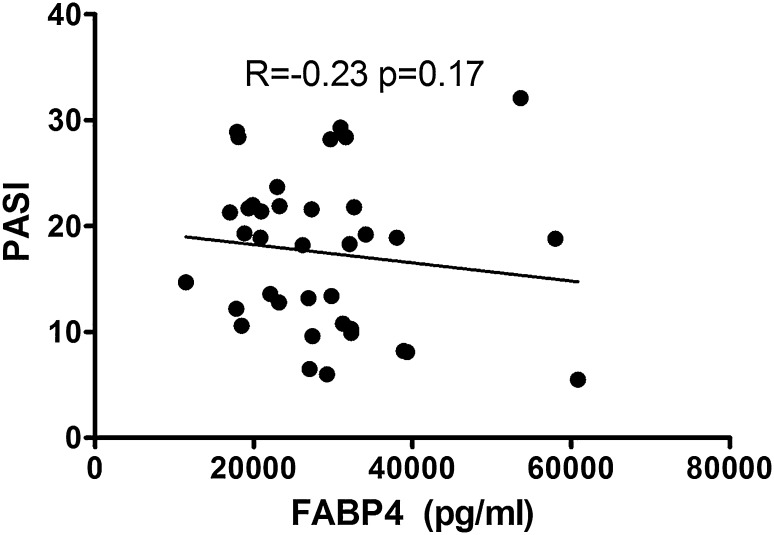
Correlation between concentrations of serum FABP4 and PASI

**Fig. 4:**
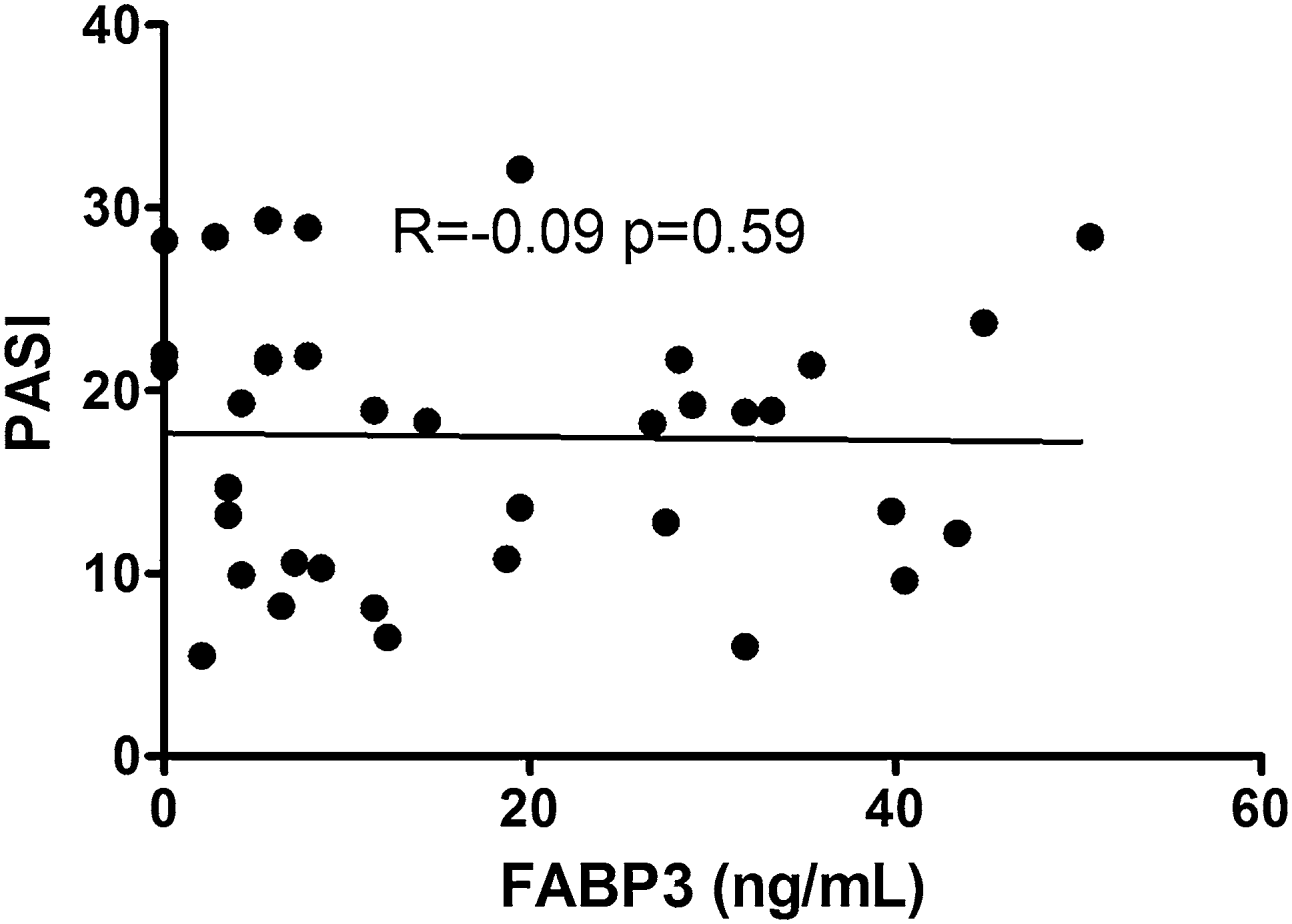
Correlation between concentrations of serum FABP3 and PASI

**Fig. 5:**
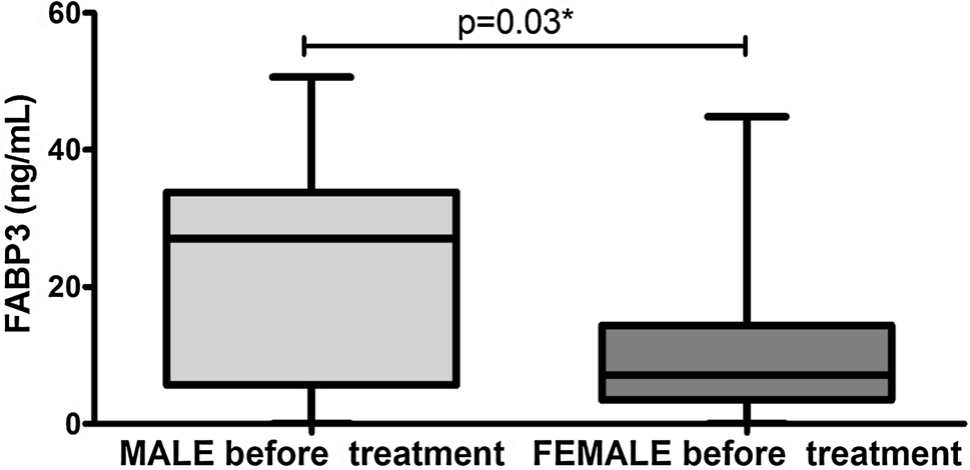
Comparison of serum FABP3 concentrations in the study group before treatment by sex

**Fig. 6:**
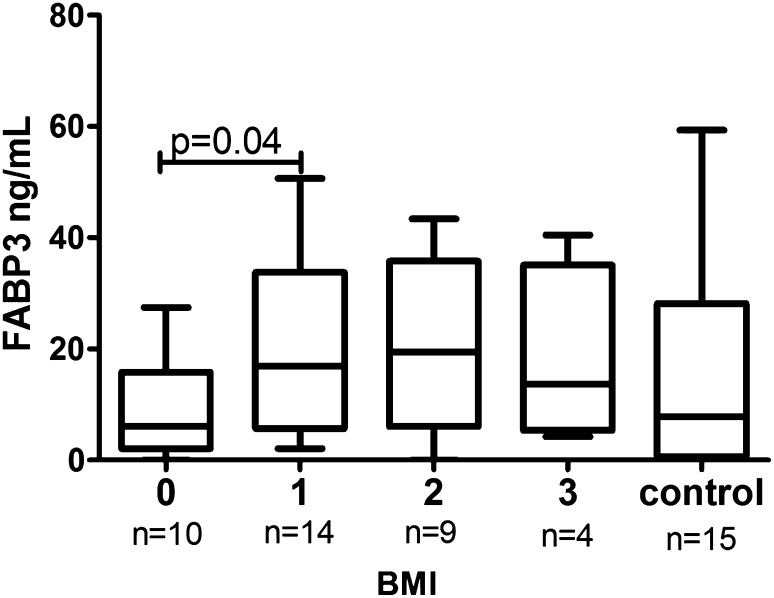
Comparison of FABP3 levels before treatment between groups of patients by BMI

**Fig. 7:**
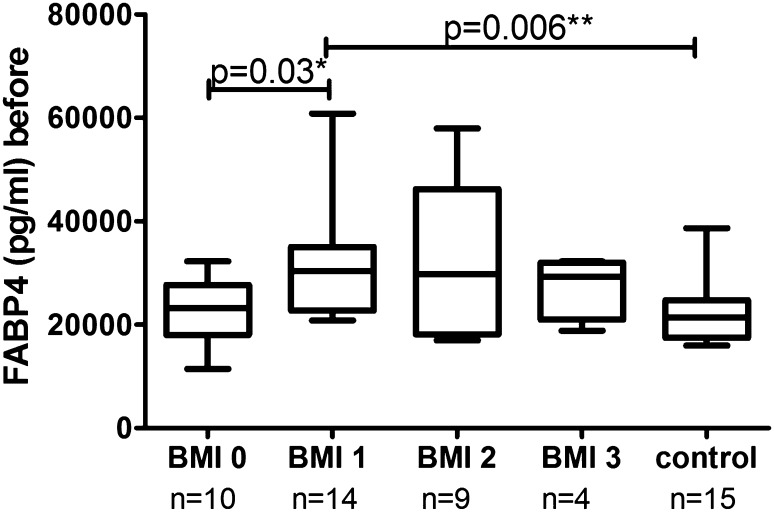
Comparison of FABP4 levels before treatment between groups of patients and controls by BMI

Clinical, demographic and laboratory data concerning the study group are summarized in Table 1. A total of 37 patients with active plaque-type psoriasis (15 women, 22 men; mean age 48.6 ± 2.4 [19–84] years) and 16 age- and sex-matched healthy individuals were enrolled in the study. Disease duration ranged from 1 to 55 years (mean, 17.4 ± 1.8 years), and the relapse duration ranged from 1 to 2 years (mean, 4.4. ± 0.9 years). The median BMI value was 27 kg/m^2^ (24–31), and the median basal PASI score was 18.8 (10.7–21.8). In the studied group, 7 patients (18.9%) had mild psoriasis (PASI <10), 16 (43.2%) had moderate disease (PASI 10–20), and 14 patients (37.8%) were diagnosed with a severe form (PASI >20). The median FABP3 concentration in psoriatic patients of 12.57 (4.23–29.6) ng/ml was not statistically different from that of healthy individuals at 7.8 (0.6–28) ng/ml (*p* = 0.47; Fig. 1). The median level of FABP3 in patients with moderate psoriasis, at 19.1 (4.5–31.1) ng/ml, was almost 2.5 times as high as that of the controls, but did not reach statistical significance (*p* = 0.19; Table 2). The lowest FABP3, 6.8 (2.1–30.0) ng/ml and slightly lower than that of the controls, was noted in the group with severe psoriasis (*p* = 0.96), but it was not statistically significant (Table 2). The median FABP4 level, 27,286 (20,344–32,257) pg/ml, was significantly higher (*p* = 0.038) than that of the controls at 21,445 (17,449–24,725) pg/ml (Fig. 2). FABP4 levels based on psoriasis activity were significantly higher in the group with PASI <10 (*p* = 0.002) compared to the controls (Table 2). Serum FABP4 and FABP3 levels showed no correlation with PASI severity score (*p* = 0.17, *p* = 0.59, respectively; Figs. 3, 4). We did not find any relation between evaluated FABP and age of the patients, or disease or relapse duration (Table 3). With regard to differences by sex in the study group, FABP3 levels were significantly higher in men than in women (*p* = 0.03; Fig. 5), with median serum FABP3 concentration in men of 27.07 (5.68–33.77) nearly four times as high as that in women, at 7.13 (3.5–14.38). In terms of links with selected proteins, a significant negative correlation was observed between FABP3 and retinol-binding protein 4 (RBP-4) (*p* = 0.02; Table 3). No links between FABP and other adipokines such as adiponectin, leptin or lipocalin-2 were noted (Table 3). In evaluating relations with basic inflammatory indices, only FABP3 and white blood cell (WBC) count showed a significant positive correlation (*p* = 0.03; Table 3). With regard to liver enzymes, FABP3 was significantly positively correlated with aspartate aminotransferase (AST) before and after treatment (*p* = 0.04, *p* = 0.025, respectively), and FABP4 with alanine aminotransferase (ALT) after treatment (*p* = 0.01; Table 3). No statistically significant correlations with FABP were noted for any of the investigated lipid parameters, either for glucose levels or BMI, aside from a significant positive relationship between FABP4 and BMI after treatment (*p* = 0.04; Table 3). We divided our psoriatic patients into four groups based on BMI, as follows: group 0, normal weight (BMI 18.5–24.9), 10 patients; group 1, overweight (BMI 25–29.9), 14 patients; group 2, obesity grade I (BMI 30–34.99), 9 patients; and group 3, obesity grade II (BMI 35–39.9), 4 patients. Serum FABP3 levels in normal-weight (group 0) patients were significantly correlated with those who were overweight (group 1; *p* = 0.04), but not with controls or other groups (Fig. 6). FABP4 concentrations in overweight individuals (BMI 25–29.9) were significantly higher than those in psoriatic patients of normal weight (*p* = 0.03) and controls (0.006; Fig. 7).

**Table 1:** Characteristics of study population (median 25–75% CI, SD, mean)

Characteristic	Psoriasis patients, *n* = 37			Control group, *n* = 16		
	Median (CI)	SD	Mean	Median (CI)	SD	Mean
SR (mm/h)	16 (7–36)	20.3	22.9	–	–	–
CRP (mg/l)	3.4 (2.1–15.6)	29	14.3	1.9 (1.2–3.0)	1.4	2.2
WBC (×10^3^/ml)	7.3 (6.3–8.7)	2.3	7.7	7.3 (6.3–8.0)	0.9	7.3
PLT (×10^3^/ml)	203 (164–265)	68.6	215	267 (249–301)	48.5	275
Glucose level	89 (77–94)	12	87	88 (80–99)	11.2	88
BMI (kg/m^2^)	27 (24–31)	5.2	28	22 (20–24)	2.7	23
Sex (male/female)	37 (22/15)			16 (7/9)		
PASI before treatment	18.8 (10.7–21.8)	7.4	17	–	–	–
PASI after treatment	8.4 (4.7–12.3)	4.4	8.4	–	–	–

**Table 2:** Serum FABP3 and FABP4 concentrations in psoriatic patients before treatment by PASI score in three groups (<10, 10–20, >20) compared to the controls

	Controls	Psoriatic patients		
		<10	10–20	>20
		*n* = 7	*n* = 16	*n* = 14
FABP3 (ng/ml)	7.8 (0.6–28)	11.5 (4.2–31.8)	19.1 (7.5–31.1)	6.8 (2.1–30.0)
*p* value (vs. controls)		0.77	0.19	0.96
FABP4 (pg/ml)	21,445 (17,449–24,725)	32,257 (27,389–39,328)	26,518 (19,332–32,205)	23,085 (19,024–31,091)
*p* value (vs. controls)		0.002*	0.14	0.35

Statistical analysis using the Mann–Whitney *U* test, median (25–75% CI)

* Statistically significant (*p* < 0.05)

**Table 3:** Main variables of the study in patients before and after treatment and correlations with serum FABP3 and FABP4 levels

Characteristic	Before treatment, *r* (*p* value)		After treatment, *r* (*p* value)	
	FABP3	FABP4	FABP 3	FABP4
SR (mm/h)	0.156 (0.36)	0.04 (0.82)	−0.197 (0.24)	0.05 (0.75)
WBC (×10^3^/ml)	0.366 (0.03)*	0.04 (0.82)	−0.191 (0.26)	0.07 (0.65)
ALT (IU/l)	0.230 (0.17)	0.14 (0.42)	−0.168 (0.32)	0.41 (0.01)*
AST (IU/l)	0.333 (0.04)*	0.15 (0.38)	0.367 (0.025)*	0.06 (0.73)
PLT (×10^3^/ml)	0.110 (0.50)	0.21 (0.22)	−0.293 (0.08)	0.15 (0.37)
BMI (kg/m^2^)	0.260 (0.12)	0.22 (0.19)	−0202 (0.23)	0.33 (0.04)*
RBC (×10^3^/ml)	0.041 (0.80)	0.01 (0.95)	0.113 (0.50)	0.10 (0.54)
Glucose level (mg/dl)	−0.0002 (0.99)	0.07 (0.68)	−0.019 (0.91)	0.14 (0.39)
CRP (mg/l)	0.049 (0.77)	0.03 (0.84)	0.065 (0.70)	0.21 (0.21)
Cholesterol (mg/dl)	0.090 (0.73)	0.09 (0.73)	0.172 (0.51)	0.03 (0.89)
HDL-C (mg/dl)	0.107 (0.68)	0.36 (0.16)	0.062 (0.81)	0.46 (0.06)
LDL-C (mg/dl)	0.065 (0.80)	0.38 (0.13)	0.054 (0.84)	−0.09 (0.71)
TAG (mg/dl)	0.054 (0.84)	0.06 (0.82)	−0.015 (0.95)	−0.29 (0.25)
Adiponectin	−0.007 (0.96)	−0.02 (0.91)	−0.114 (0.50)	−0.04 (0.77)
Leptin	0.004 (0.99)	0.11 (0.51)	0.227 (0.17)	0.16 (0.32)
RBP-4	0.067 (0.69)	−0.38 (0.02)*	−0146 (0.38)	−0.35 (0.03)*
FABP3	–	0.15 (0.37)	–	0.29 (0.08)*
FABP4	0.150 (0.37)	–	0.29 (0.08)*	–
Age (years)	−0.001 (0.99)	−0.07 (0.67)	−0.040 (0.813)	0.05 (0.75)
Relapse duration (months)	0.170 (0.31)	0.18 (0.29)	0.244 (0.15)	−0.01 (0.92)
Disease duration (years)	−0.217 (0.20)	−0.15 (0.39)	0.202 (0.23)	0.02 (0.93)

* Statistically significant correlation (*p* < 0.05), *p* values obtained by Spearman correlation

**Fig. 8:**
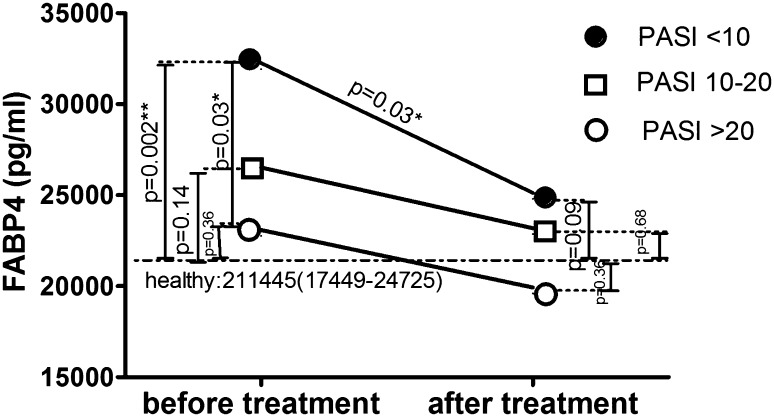
Comparison of serum FABP4 concentrations between patients and controls before and after treatment, by PASI score

After 14 days of topical therapy with 5% salicylic acid ointment and 0.3% anthralin, skin lesions in all patients improved. The median total PASI score after therapy decreased to 8.4 (4.7–12.3) from the basal PASI of 18.8 (10.7–21.8; Table 1). There was no change in median FABP3 levels after therapy (*p* = 0.5), with levels after treatment remaining comparable to those before treatment and to those of the controls (Table 4). Similar to the analysis of the whole study population, the assessments carried out among groups with different disease severity showed no significant influence of therapy on serum FABP3 concentrations (Table 5). There was no meaningful change in median FABP4 concentration after topical therapy. However, there was a non-significant reduction (*p* = 0.61) relative to controls, losing statistical significance from before treatment (*p* = 0.038; Table 4). In patients with PASI <10, serum FABP4 levels were significantly decreased from levels before treatment (*p* = 0.03; Table 5). In patients with mild psoriasis, FABP4 levels were significantly higher than those in controls before treatment (*p* = 0.002), but the differences did not remain significant after therapy (*p* = 0.09; Fig. 8). There were no statistically significant differences in serum FABP3 or FABP4 levels after treatment based on severity of psoriasis (Table 6).

**Table 4:** Serum FABP3 and FABP4 concentrations in psoriatic patients before and after treatment compared to the control group

	Controls	Psoriatic patients	
		Before treatment	After treatment
FABP3 (ng/ml)	7.86 (0.61–28.16)	11.48 (4.96–30.33)	12.93 (3.51–30.33)
*p* value (vs. controls)		0.47	0.50
FABP4 (pg/ml)	21,445 (17,449–24,725)	27,286 (20,344–32,257)	23,034 (18,320–29,874)
*p* value (vs. controls)		0.038*	0.614

Statistical analysis using the Mann–Whitney *U* test, median (25–75% CI)

* Statistically significant (*p* < 0.05)

**Table 5:** Serum FABP3 and FABP4 concentrations in psoriatic patients before and after treatment, by PASI score in three groups (<10, 10–20, >20) in relation to the controls

	PASI		
	<10	10–20	>20
FABP3 (ng/ml)			
Before treatment	11.5 (4.2–31.8)	19.1 (7.5–31.1)	6.8 (2.1–30.0)
After treatment	10.0 (0.6–15.8)	11.5 (3.5–32.5)	20.5 (7.8–34.7)
*p* (before vs. after treatment)	0.62	0.36	0.22
FABP4 (pg/ml)			
Before treatment	32,257 (27,389–39,328)	26,518 (19,332–32,205)	23,085 (19,024–31,091)
After treatment	25,134 (22,777–27,082)	23,187 (18,038–35,139)	19,934 (16,513–27,735)
*p* (before vs. after treatment)	0.03*	0.65	0.36

Statistical analysis using the Mann–Whitney *U* test

* Statistically significant (*p* < 0.05)

**Table 6:** Serum FABP3 and FABP4 concentrations in psoriatic patients after treatment, by PASI score (<10, 10–20, >20)

	PASI		
	<10 vs. 10–20	<10 vs. >20	10–20 vs. >20
FABP3	0.48	0.11	0.32
FABP4	0.55	0.11	0.60

Statistical analysis using the Mann–Whitney *U* test

Significance level *p* < 0.05

## Discussion

It is extremely difficult to discuss the results obtained in this work when, to the best of our knowledge, there is not a single study in the published literature regarding the role of the heart and adipocyte isoforms of FABP in psoriasis, or the relationship with antipsoriatic therapy. A few papers have shown only the importance of FABP5 in psoriatic keratinocyte differentiation proving disturbances in fatty acid metabolism in the lesions but also in the uninvolved skin of patients with psoriasis [13, 22]. Miyake *et al* evaluated FABP5 levels in serum and tape-stripped psoriatic skin [22]. Serum FABP5 levels were similar between patients with psoriasis and controls, and did not reflect the skin condition. Levels of FABP5 were statistically elevated in psoriatic lesions compared with uninvolved skin. Skin-stripping FABP5 did not correlate with PASI, but was statistically associated with total erythema, induration and desquamation scores. The authors suggested that skin-stripping FABP5 levels are related instead to local skin condition. Miyake *et al*. also investigated FABP5 (E-FABP) during anti-TNF-α and narrow-band ultraviolet B (NB-UVB) light therapy. They noted a reduction in skin-stripping FABP5 levels and posited that it could be used to monitor therapy outcome [22]. It should be noted, however, that these results were obtained in a very small sample—seven patients—treated with different methods (two patients with adalimumab, three with infliximab and two with NB-UVB), rendering them very speculative.

A study by Kucharekova *et al*. failed to demonstrate upregulation of FABP5 after single use of anthralin on uninvolved psoriatic skin, thus refuting the thesis regarding the function of the protein in oxidative stress or skin barrier impairment caused by the topical agent [23]. In our study, we evaluated the influence of a longer course of therapy with anthralin, but on other FABP isoforms and their levels in serum, not in the skin.

Considering that FABP3 is a highly sensitive predictor of MI, especially in patients with MS, which is strongly linked to psoriasis, we assumed that this protein could serve as a marker of heart failure in patients with this dermatosis. Unfortunately, there was no change in serum FABP3 levels in our patients compared to the healthy individuals. However, the concentration was almost 2.5 times as high in persons with PASI 10–20 as in controls, indicating that FABP3 could have some relevance in patients with moderate psoriasis. The significant positive correlation between FABP3 and WBC leads us to speculate that this protein could be an indicator of inflammation in psoriatic patients. However, no relationship with CRP was shown. A statistically significant association between FABP3 and AST activity points to some unknown link with liver function or perhaps non-alcoholic fatty liver disease (NAFLD) and psoriasis. On the other hand, the liver is involved in metabolic processes and lipid metabolism, and our study did not show any relationship between FABP3 and indicators of metabolic disorders such as glucose level, BMI, lipid profile or other adipokines involved in the pathogenesis of many common systemic diseases including psoriasis. Analysis of demographic features including age and the duration of the disease and relapse revealed no statistical correlation with FABP3 or FABP4. We noted significantly higher levels of FABP4 in men with psoriasis compared to women. Pelsers *et al*. also found higher concentrations of the heart isoform of FABP in men as well as an increase with age, but in healthy volunteers [24]. Conversely, FABP4 seems to be more closely related to women and age, but it has not been investigated in relation to psoriasis [18, 25]. FABP4 was not associated with sex in our study group.

Although FABP4 is the most commonly known isoform of FABP, no studies have investigated its role in psoriasis. This adipokine has been linked to IR, DM, MS, obesity, atherosclerosis, CAD and NAFLD. We can speculate that FABP4 may be a link between psoriasis and its metabolic comorbidities, especially because we found significantly increased serum concentration of this adipokine. Thus FABP4 could be a biomarker of psoriasis or a predictor of metabolic disorders and their complications in our patients, particularly those with a mild type of psoriasis. In terms of links between inflammatory or metabolic indicators and FABP4, we found no correlation with WBC, CRP or glucose levels aside from a positive relationship with BMI, which was significant only after therapy. Our results showed that serum FABP4 concentration was significantly higher in overweight patients than in normal-weight psoriatic patients or in controls. Surprisingly, adipokine levels did not correlate with BMI or adipose tissue content expressed as weight. Therefore, it must be assumed that serum FABP4 levels in psoriatic patients are dependent not only on the adipose tissue content expressed by BMI, for example, but also on various other modified effectors influencing different inflammatory or immunological stimuli in psoriasis. As for the lipid profile, we did not observe statistical correlations. Several studies have noted positive correlations between FABP4 and high-density lipoprotein (HDL) and low-density lipoprotein (LDL) cholesterol, triacylglycerol (TAG), BMI, CRP or adiponectin and TNF-α [14, 18, 26]. Zhang *et al*. did not prove a positive link with BMI in individuals with chronic obstructive pulmonary disease [25]. With respect to the relationship with other adipokines, no association was demonstrated in the present study between adiponectin and leptin, other than a significant inverse relationship with RBP-4. In our previous work, we hypothesized that RBP-4 might have a protective role in terms of chronic inflammation and comorbidities of psoriasis [7]. This adipokine has been associated in particular with IR, DM, obesity and MS or cardiometabolic risk, which are relevant in psoriasis [27]. With regard to the negative relation between RBP-4 and FABP4, we can assume that the latter could serve as an indicator of comorbidities in psoriatic patients, as its level was significantly increased in the study group.

In our study, after 2 weeks of topical treatment with keratolytic ointment and anthralin, we found no significant influence on median serum levels of FABP in psoriatic patients compared to healthy individuals. However, in relation to the level of psoriasis activity, FABP4 was decreased, losing its basal significantly higher level in comparison to the controls. With regard to the impact of topical therapy on FABP levels by PASI score, FABP4 in patients with mild psoriasis lost its basal significance, but the level did not reach statistical significance. We can assume that, even though anthralin may be considered an outdated treatment, it may influence lipid metabolism through its impact on FABP4 in patients with psoriasis. The adipokine could be useful in determining the effectiveness of anthralin in psoriatic patients with a mild type of the disease.

Because FABP are responsible for the integration of inflammatory or metabolic processes and are strongly involved in the development of MS, IR, CAD and NAFLD, they have become a therapeutic target. Several studies have reported the efficacy of synthetic FABP4 inhibitors against the diseases mentioned above [6, 10, 14]. Good therapeutic effects by reducing FABP4 levels were noted with the use of BMS309403 FABP4-neutralizing antibodies as well as other commonly used drugs such as sitagliptin, angiotensin II receptor blockers and omega-3 fatty acids [10, 28–30]. These promising data enable us to draw conclusions regarding the potential use of drugs reducing FABP4 levels also for protecting patients with psoriasis from cardiovascular or metabolic disorders. Looking at the possible medical insights concerning FABP, more research is needed to clarify the role of FABP in psoriasis or its neutralizing antibodies in future antipsoriatic therapy. There are some limitations of this study that must be considered when analyzing the data presented. The major limitation is the small number of patients (*n* = 37), although they were evaluated at two time points. The number of patients grouped by disease severity (PASI 1 *n* = 7, PASI 2 *n* = 16, PASI 3 *n* = 14) is also inadequate. A larger group with mild psoriasis is definitely advisable for future investigations. In addition, the designation of FABP4 is indicated for comparison with other FABP assessed in the study. We plan to extend our investigation to a greater number of patients, different methods of treatment and further isoforms of FABP in subsequent stages of our research.

Serum FABP4 levels were significantly increased in patients with psoriasis, indicating that this protein may be a marker of psoriasis and an independent predictor of the risk of comorbidities or complications in psoriatic patients. The FABP studied in this work do not appear to be useful for determining the severity of psoriasis. A positive correlation between FABP3 and white blood count or liver enzyme activity points to a possible indication of inflammation or a link between psoriasis and liver disorders. The evaluated proteins may not be helpful for monitoring the efficacy of standard topical treatment. In conclusion, data from the literature are sparse and inconsistent, and they refer to patients with diseases other than psoriasis, so it is impossible to clearly interpret our findings. However, the above-mentioned limitations of this study prevent us from drawing definitive conclusions regarding the role of FABP3 and FABP4 as predictive factors for psoriatic patients. Nevertheless, it underscores the uniqueness of the present study and the need for further study to determine the precise role of FABP4 and FABP3 in the pathogenesis of psoriasis and its comorbidities.

## Abbreviations

aP2: Adipocyte protein 2
BMI: Body mass index
CAD: Coronary artery disease
CRP: C-reactive protein
DM: Diabetes mellitus
ELISA: Enzyme-linked immunosorbent assay
FA: Fatty acids
FABP: Fatty acid-binding proteins
FABP1: Liver fatty acid-binding protein
FABP3: Heart fatty acid-binding protein
FABP4: Adipocyte fatty acid-binding protein
FABP5: Epidermal fatty acid-binding protein
HDL-C: High-density lipoprotein cholesterol
IR: Insulin resistance
L-FABP: Liver fatty acid-binding protein
LCFA: Long-chain fatty acids
LDL-C: Low-density lipoprotein cholesterol
MI: Myocardial infarction
MS: Metabolic syndrome
NAFLD: Non-alcoholic fatty liver disease
NB-UVB: Narrow-band ultraviolet B
PA-FABP: Psoriasis-associated fatty acid-binding protein
PASI: Psoriasis Area and Severity Index
PLT: Platelets
RBC: Red blood count
RBP-4: Retinol-binding protein 4
SR: Sedimentation rate
TAG: Triacylglycerol
TNF-α: Tumor necrosis factor-α
VEGF: Vascular endothelial growth factor
WBC: White blood cell
